# Parental knowledge and attitudes to childhood hearing loss and hearing services in Qassim, Saudi Arabia

**DOI:** 10.1186/s12887-020-02080-2

**Published:** 2020-04-20

**Authors:** Ali Mohammed Alsudays, Abdulmajeed Abdullah Alharbi, Faris Saleh Althunayyan, Abdulrahman Abdullah Alsudays, Sultan Mohammed Alanazy, Osama Al-Wutay, Mazyad Marji Alenezi

**Affiliations:** 1 Otolaryngology Head and Neck Surgery Resident, Prince Sultan Military Medical City, Riyadh, Kingdom of Saudi Arabia; 2 Department of Dermatology, King Saud Hospital, Qassim, Kingdom of Saudi Arabia; 3 General Pediatrics Resident, King Abdulaziz Medical City, Riyadh, Kingdom of Saudi Arabia; 4 Otolaryngology Head and Neck Surgery, Department of Surgery, Unaizah College of Medicine and Medical Sciences, Qassim University, Buraydah, Kingdom of Saudi Arabia; 5 Community Medicine, Department of Family and Community Medicine, Unaizah College of Medicine and Medical Sciences, Qassim University, Buraydah, Kingdom of Saudi Arabia; 6 Otolaryngology Head and Neck Surgery, College of Medicine, Qassim University, Buraydah, Kingdom of Saudi Arabia

**Keywords:** Knowledge, Attitude, Childhood, Hearing loss, Hearing services, Saudi Arabia

## Abstract

**Background:**

Successful audiology service delivery depends on support from the community, and agreement to utilize hearing healthcare programs. Assessment of parents’ awareness regarding hearing loss (HL) and audiology services is necessary for the development of suitable hearing programs for children. Previous studies reported that early detection and intervention for hearing problems are typically strongly supported by parents. The current study sought to evaluate parents’ knowledge and attitudes regarding childhood HL and hearing services.

**Methods:**

A cross-sectional study conducted at five centers in Qassim region of Saudi Arabia. A self-report questionnaire was administered to collect demographic data in addition to 31 questions regarding the knowledge and attitudes of parents toward HL. IBM SPSS Statistics for Windows, Version 21 was used for data analysis. A *p*-value cut-off point of 0.05 at 95% CI was used to determine statistical significance. The analyses examined the association between socio-demographic characteristics and knowledge and attitudes toward HL using chi-square tests.

**Results:**

Overall, participants included in this study were 243 participants. Of these, 105 (43.2%) were fathers, and 138 (56.8%) were mothers. Ages ranged from 21 to 60+ years. Assessment of the prevalence of various aspects of knowledge and attitudes among parents toward childhood HL revealed that 103 participants (42.4%) possessed good knowledge, while 140 participants (57.6%) possessed poor knowledge. In contrast, the attitude analysis revealed that 224 participants (92.2%) expressed positive attitudes, while only 19 participants (07.8%) showed a negative attitude regarding audiology services. We found a significant association between age group and knowledge (*p* = 0.002).

**Conclusion:**

Most parents in our sample possessed poor knowledge regarding childhood HL. However, most parents expressed positive attitudes regarding audiology services. The current findings suggest a need to increase awareness among parents regarding childhood HL.

## Background

Audiology services depend on support from the community, and agreement to utilize hearing healthcare programs [1]. The assessment of parents’ awareness regarding hearing loss (HL) and audiology services is essential for developing suitable and comprehensive hearing programs for children [2]. Previous studies reported that early detection and intervention for hearing problems are typically strongly supported by parents [3–6]. To ensure that children with hearing difficulties reach a normal level of language and speech ability compared with their healthy peers, a program called Universal Newborn Hearing Screening (UNHS) has been implemented in several countries, enabling the diagnosis and management of hearing difficulties in early childhood [7]. Parents’ knowledge has been found to play a crucial role in determining the success of UNHS, and parents with low levels of knowledge tend to have less positive attitudes toward such programs [8]. In the past decade, infants with permanent childhood HL were able to be identified at an earlier stage of life due to the increased implementation of such programs [9]. There is evidence that these programs can substantially reduce or prevent the impact of sensorineural HL (SNHL) on speech and language learning [10, 11].

Many previous studies have been conducted to evaluate knowledge regarding HL. First, an interview-based study in India evaluated grandmothers’ knowledge about HL in India. They found that most participants were aware that HL can be congenital (63%), due to noise exposure (62%) or as a result of ear discharge (61%). However, other causes were not known by more than 50% of the participants. In addition, only 20% of participants knew that HL can be diagnosed at birth, and only 12% of grandmothers were aware of screening programs for newborns [4]. In China, a study regarding infant HL was performed with 115 mothers. Participants were aware that environmental noise, ear discharge, and high fever could act as predisposing factors for the development of HL. However, the results revealed poor knowledge regarding jaundice, measles, and convulsion as risks for HL. 99% of participants reported that they would screen their babies after birth if the service was available [12]. Another study examined 100 mothers and 50 fathers, who completed a questionnaire in in-person interviews. The results revealed that otitis media (OM) was the best known predisposing factor for childhood HL (94%), followed by noise exposure (87.3%), and family history (72.7%). Parental awareness of common childhood immunizations was highest compared with other public health initiatives to prevent/reduce OM (84%), followed by breast-feeding (76%).

Fathers had higher rates of awareness than mothers, including OM (*p* = 0.038), breast-feeding (*p* = 0.031) and noise exposure (*p* = 0.007). Almost half of the parents tested (56%) reported the belief that supernatural curses can cause HL. Positive support regarding screening programs for infant hearing was conveyed in most parental responses (96%), as was support for school-based hearing and ear health observations and examinations (99.3%) [13]. Another study regarding the hearing services showed that the attitude and practices toward hearing services for the children were highly positive. The acceptance of newborn hearing tests reaches 96% of the parents, OAE accepted by 93.3, 94% of the parents were accepting the use of hearing aids if needed and the surgery the correct the hearing accepted by 64% of the parents [14]. There are multiple factors that increase parental knowledge and attitude towards hearing loss and hearing services, which include older age group, female gender, high income, and high level of education [15–17]. The poor knowledge of the parents can lead to late diagnosis and treatment, so the current study is trying to assess the knowledge and attitude regarding hearing loss and hearing services.

### Objective

The current study sought to evaluate parents’ knowledge and attitudes regarding childhood HL and hearing services.

## Methods

A cross-sectional study was conducted at five centers in Qassim region of Saudi Arabia. We included all parents whose children were attending well baby and ENT clinics between August 26th 2018 and September 6th 2018 at five medical centers. We targeted all the parents regardless of having a child with hearing loss or not as we want to assess the knowledge and attitude of the general population. In addition, based on our culture and beliefs, we assume there might be a difference between fathers and mothers, so we compared them. The study received ethical approval from the ministry of health in Saudi Arabia. We administered a self-report questionnaire to collect demographic data in addition to 31 questions about the knowledge and attitudes of parents toward HL. This questionnaire adopted from the Year 2007 Position Statement and Kaspar A study [14]. Most risk factors included in the questionnaire adapted from the risk indicators of Year 2007 Position Statement [18]. A pilot study was conducted to ensure clarity and convenience of the questions and to estimate the time needed to fill the questionnaire.

### Data analysis

SPSS version 21 was used for data analysis. A *p*-value cut-off point of 0.05 at 95% CI used to determine statistical significance. The analyses examined the association between socio-demographic characteristics and knowledge and attitudes toward HL using chi-square tests.

In the section of the questionnaire evaluating knowledge and attitudes about childhood HL presented in Table 2, knowledge was assessed with 25 questions, and attitudes were assessed with six questions. The response options were “no”, “yes” and “don’t know”. Points were allocated to the selected option, with 1 point for “yes”, while “no” and “don’t know” were given 0 points. A final score ranging from 0 to 25 points was calculated for each participant in the knowledge evaluation. Based on the final score, the level of knowledge was classified into “poor knowledge” for scores of 0–12 points, and “good knowledge” for scores of 13–25 points. Attitude was scored from 0 to 6 points. Attitude was considered to be negative for scores of 0–3 points and positive for scores of 4–6 points.

## Results

A total of 243 participants were included in this study. The overall response rate of participants was 90.67%, with complete responses to 243 of the 268 distributed questionnaires. Of the 243 participants, 105 (43.2%) were fathers, and 138 (56.8%) were mothers. Ages ranged from 21 to 60+ years, with 49 participants (20.2%) in the 21–30 years age group, 86 participants (35.4%) in the 31–40 years age group, 67 participants (27.6%) in the 41–50 years age group, 27 participants (11.1%) in the 51–60 years age group, and 14 participants (05.8%) in the 60+ years age group. In addition, 45.7% of participants were university graduates, while 54.3% of participants received secondary school education or below as shown in Table 1.

**Table 1:** Parents’ demographic data

Study variables	Father N (%) (***n*** = 105)	Mother N (%) (***n*** = 138)	Overall N (%) (***n*** = 243)
**Age group**			
• 21–30	12 (11.4%)	37 (26.8%)	49 (20.2%)
• 31–40	28 (26.7%)	58 (42.0%)	86 (35.4%)
• 41–50	41 (39.0%)	26 (18.8%)	67 (27.6%)
• 51–60	15 (14.3%)	12 (08.7%)	27 (11.1%)
• > 60	09 (08.6%)	05 (03.6%)	14 (05.8%)
**Level of education**			
• Secondary or below	52 (49.5%)	80 (58.0%)	132 (54.3%)
• University	53 (50.5%)	58 (42.0%)	111 (45.7%)

The knowledge and attitudes of parents regarding childhood HL are shown in Table 2. Both fathers and mothers exhibited a relatively high level of knowledge relation to the statement “children with HL can attend school”, followed by the statement “babies can be born with HL”. The results revealed that the lowest level of knowledge among both fathers and mothers was in relation to the statement “jaundice can cause HL”, followed by “low birth weight <1500 g can cause HL.” Regarding attitudes toward childhood audiology services, mothers expressed more positive attitudes than the fathers in all attitude questions.

**Table 2:** Summary of parental knowledge and attitudes to childhood hearing loss and hearing services

Statement	FATHER (n = 105)			MOTHER (n = 138)		
	YES (%)	NO (%)	Don’t know (%)	YES (%)	NO (%)	Don’t know (%)
**Knowledge: SNHL risk factors**						
1. Babies can be born with HL	72.4%	06.7%	21.0%	79.7%	06.5%	13.8%
2. CNS infection can cause HL	53.3%	03.8%	42.9%	55.1%	03.6%	41.3%
3. Neonatal infection can cause HL	36.2%	17.1%	46.7%	34.1%	20.3%	45.7%
4. Maternal infection can cause HL	36.2%	17.1%	46.7%	26.1%	24.6%	49.3%
5. Drugs/medications can cause HL	38.1%	20.0%	41.9%	48.6%	15.9%	35.5%
6. Radiotherapy can cause HL	32.4%	15.2%	52.4%	46.4%	16.7%	37.0%
7. Chemotherapy can cause HL	39.0%	11.4%	49.5%	33.3%	18.1%	48.6%
8. Jaundice can cause HL	13.3%	41.9%	44.8%	13.8%	60.1%	26.1%
9. Delayed crying at birth can cause HL	41.9%	28.6%	29.5%	34.1%	52.9%	13.0%
10. Family history can cause HL	35.2%	42.9%	21.9%	44.9%	38.4%	16.7%
11. Consanguineous marriage can cause HL	44.8%	37.1%	18.1%	47.8%	41.3%	10.9%
12. Low birth weight < 1500 g can cause HL	14.3%	34.3%	51.4%	18.1%	57.2%	24.6%
13. CHL/SNHL risk related	47.6%	24.8%	27.6%	32.6%	40.6%	26.8%
14. Craniofacial anomalies can cause HL	47.6%	14.3%	38.1%	49.3%	22.5%	28.3%
15. Head trauma can cause HL	71.4%	06.7%	21.9%	66.7%	13.0%	20.3%
**Non-biomedical model beliefs**						
16. Evil spirits can cause HL	41.0%	22.9%	36.2%	38.4%	25.4%	36.2%
17. Curses can cause HL	58.1%	14.3%	27.6%	52.2%	18.8%	29.0%
**Knowledge: OM and CHL risk factors**						
18. Ear discharge and OM can cause HL	61.0%	12.4%	26.7%	60.1%	21.7%	18.1%
19. Recurrent URTI can cause OM	42.9%	25.7%	31.4%	44.9%	38.4%	16.7%
20. Breast-feeding for first 6 months reduce/prevent OM	48.6%	16.2%	35.2%	50.7%	34.1%	15.2%
21. Smoking can predispose to OM	20.0%	40.0%	40.0%	18.1%	59.4%	22.5%
22. Routine childhood immunizations can reduce OM	49.5%	22.9%	27.6%	42.0%	28.3%	29.7%
**Knowledge: Identification and intervention**						
23. HL can be identified soon after birth	52.4%	29.5%	18.1%	52.9%	34.8%	12.3%
24. Treatment for HL is available	59.0%	12.4%	28.6%	63.8%	14.5%	21.7%
25. Children with HL can attend school	77.1%	09.5%	13.3%	83.3%	09.4%	07.2%
**Attitudes toward childhood audiology services**						
1. I would like my baby tested soon after birth	81.9%	13.3%	04.8%	90.6%	04.3%	05.1%
2. I would accept OAE hearing screening test for my baby	84.8%	07.6%	07.6%	94.2%	02.9%	02.9%
3. I would like my child tested at school	87.6%	08.6%	03.8%	94.9%	03.6%	01.4%
4. I would let my child use hearing aids	84.8%	09.5%	05.7%	88.4%	07.2%	04.3%
5. I would accept ear surgery for my child	88.6%	01.0%	10.5%	89.9%	03.6%	06.5%
6. I would like more information	88.6%	03.8%	07.6%	92.0%	06.5%	01.4%

*SNHL* sensorineural hearing loss, *CNS* central nervous system, *HL* hearing loss, *OM* otitis media, *OAE* oto-acoustic emission

The overall level of parental knowledge and attitudes is shown in Table 3. The results revealed that 103 participants (42.4%) possessed good knowledge about childhood HL, while 140 participants (57.6%) exhibited poor knowledge. However, 224 participants (92.2%) expressed positive attitudes toward childhood HL, while only 19 participants (7.8%) expressed negative attitudes.

**Table 3:** The level of parental knowledge and attitudes

Level of knowledge and attitudes	N (%) (n = 243)
Knowledge	
• Good	103 (42.4%)
• Poor	140 (57.6%)
Attitude	
• Positive	224 (92.2%)
• Negative	19 (07.8%)

The relationships between knowledge, attitudes, and socio-demographic characteristics of parents are shown in Table 4, with *p*-values indicating that the relationship was statistically significant. The results revealed a significant association between age group and knowledge (*p* = 0.002) but no significant relationship with attitude.

**Table 4:** Relationship between knowledge, attitudes, and socio-demographic characteristics of parents

Factor	Knowledge		Attitude	
	Good N (%) (***n*** = 103)	Poor N (%) (***n*** = 140)	Positive N (%) (***n*** = 224)	Negative N (%) (***n*** = 19)
**Age group**				
• 21–30	13 (12.6%)	36 (25.7%)	45 (20.1%)	04 (21.1%)
• 31–40	30 (29.1%)	56 (40.0%)	77 (34.4%)	09 (47.4%)
• 41–50	34 (33.0%)	33 (23.6%)	63 (28.1%)	09 (47.4%)
• 51–60	16 (15.5%)	11 (07.9%)	25 (11.2%)	02 (10.5%)
• > 60	10 (09.7%)	04 (02.9%)	14 (06.2%)	0
***P-value***	0.002 *		***0.671***	
**Parents’ gender**				
• Father	45 (43.7%)	60 (42.9%)	93 (41.5%)	12 (63.2%)
• Mother	58 (56.3%)	80 (57.1%)	131 (58.5%)	07 (36.8%)
***P-value***	***0.897***		***0.068***	
**Level of education**				
• Secondary school or below	56 (54.4%)	76 (54.3%)	120 (53.6%)	12 (63.2%)
• University	47 (45.6%)	64 (45.7%)	104 (46.4%)	07 (36.8%)
***P-value***	***0.990***		***0.421***	

*P*-values were calculated using chi-square tests. *Significant at *p* ≤ 0.05 level

In addition, no significant relationships were found between knowledge or attitude and gender or level of education.

## Discussion

HL can be a substantial barrier to education and social integration. Early identification and intervention for HL can provide important benefits because hearing is critical for learning oral communication, as well as academic and social participation [19]. The current findings revealed that both fathers and mothers possessed a relatively high level of knowledge in relation to the statement “babies can be born with HL”, followed by the statement “head trauma can cause HL”, but possessed the least knowledge in relation to the statement “jaundice can cause HL”, followed by the statement “low birth weight <1500 g can cause HL.” A previous study by Ravi et al. reported that knowledge of risk factors among participants was higher in relation to the statement “prolonged noise can cause HL”, while participants possessed poor knowledge regarding the statement “drugs/medication can cause HL.” [5] In contrast, a study of knowledge on SNHL by Kaspar and colleagues revealed that participants had better knowledge about “noise exposure” and “family history”, but poor knowledge regarding the statement “jaundice can cause HL”, followed by “delayed crying at birth can cause HL.” [14] In contrast, Sanju and colleagues reported that, among a sample of surveyed nurses, 70% were aware of the “harmful effect of noise on hearing to infant” while most were not aware of the consequences of “hyperbilirubinemia on infants hearing.” [20] The aspects of knowledge about risk factors of HL highlighted in previous studies differed from the results in the current study. Moreover, the current findings suggest differences in participants’ knowledge regarding HL, compared with previous studies. There are no similar studies done in the region regarding this topic which may contribute to these discrepancies may have also been caused by differences in the culture and believes among the people in Saudi Arabia.

Moreover, questions about the knowledge of parents regarding OM in the current study revealed that both fathers and mothers possessed a relatively high level of knowledge in relation to the statement that “ear discharge and OM can cause HL”, whereas questions about the identification and intervention of HL and revealed a high level of knowledge in relation to the statement “children with HL can attend school.” These findings are consistent with those of previous studies conducted in India and Solomon Island [4, 5]. Moreover, a low level of knowledge in relation to the statement “treatment for hearing loss is available” was also highlighted in both studies.

Regarding the attitudes and practices of parents in relation to childhood audiology services, parents typically expressed positive attitudes in relation to the statement “I would like my child tested at school” followed by “more information about the service.” This finding conflicts with the results of previous studies in India and Solomon island [5, 14], but further confirmed the positive attitudes of parents toward childhood audiology services. In contrast, Sanju and colleagues reported that 40% of surveyed nurses expressed negative attitudes in relation to the statement “HL can be identified soon after birth”, and 88% of nurses were unaware that some children with hearing impairments can still hear and speak [20].

The current study also examined the overall knowledge and attitudes of parents toward childhood HL, revealing that 103 parents (42.4%) possessed good knowledge, while 140 parents (57.6%) possessed poor knowledge. However, 224 parents (92.2%) expressed positive attitudes toward childhood audiology services, with only 19 parents (07.8%) expressing negative attitudes. To our knowledge, this is the first study to calculate the overall prevalence of knowledge and attitudes toward childhood HL in Saudi Arabia. Overall, our findings revealed that most participants possessed poor knowledge regarding childhood HL, although most expressed positive attitudes and practices regarding the hearing tests and audiology services.

Moreover, we also measured the relationship between knowledge, attitudes, and socio-demographic characteristics of parents, revealing a significant relationship between age group and knowledge level as we have found out that people above the age of 40 years old have good knowledge towards children hearing loss in comparison to those who are below the age of 40 years old. To our knowledge, this is the first study to report this association.

## Conclusion

Most parents in this study possessed poor knowledge regarding childhood HL, but expressed positive attitudes regarding audiology services. These findings suggest the need to increase awareness about childhood HL among parents.

Our main target is parents, so we will start by publishing a brochure about children hearing loss and its risk factors in hospitals, primary health care centers, schools, and universities. Moreover, we will increase the awareness of the community toward this subject by making lots of campaigns about children hearing loss.

## Data Availability

The datasets used and/or analysed during the current study are available from the corresponding author on reasonable request.

## Abbreviations

HL: Hearing loss
UNHS: Universal Newborn Hearing Screening
SNHL: Sensorineural hearing loss
OM: Otitis media
ENT: Ear nose and throat
OAE: Otoacoustic Emissions Test

## References

[1] Desgeorges J (2003). Family perceptions of early hearing, detection, and intervention systems: listening to and learning from families. Ment Retard Dev Disabil Res Rev.

[2] Matt SB. Perceptions of disability among parents of children with disabilities in Nicaragua: Implications for future opportunities and health care access. Disabil Stud Q. 2014;34(4) [Accessed 2018 May 21]. Available from: http://dsq-sds.org/article/view/3863.

[3] Olusanya BO, Luxon LM, Wirz SL (2006). Maternal views on infant hearing loss in a developing country. Int J Pediatr Otorhinolaryngol.

[4] Selvarajan H, Rajendran A, Ninan B, Rajagopalan R (2014). Grandmothers’ perspective on hearing loss in children and newborn hearing screening. Indian J Otol.

[5] Ravi R, Yerraguntla K, Gunjawate DR, Rajashekhar B, Lewis LE, Guddattu V (2016). Knowledge and attitude (KA) survey regarding infant hearing loss in Karnataka, India. Int J Pediatr Otorhinolaryngol.

[6] Swanepoel D, Almec N (2008). Maternal views on infant hearing loss and early intervention in a South African community. Int J Audiol.

[7] Durieux-Smith A, Fitzpatrick E, Whittingham J (2008). Universal newborn hearing screening: a question of evidence. Int J Audiol.

[8] Weichbold V, Welzl-Müller K (2000). Universelles Neugeborenen-Hörscreening - Einstellung und Ängste der Mütter. HNO..

[9] Vohr BR, Carty LM, Moore PE, Letourneau K (1998). The Rhode Island hearing assessment program: experience with statewide hearing screening (1993-1996). J Pediatr.

[10] Moeller MP (2000). Early intervention and language development in children who are deaf and hard of hearing. Pediatrics..

[11] Yoshinaga-Itano C, Sedey AL, Coulter DK, Mehl AL (1998). Language of early- and later-identified children with hearing loss. Pediatrics..

[12] Wang X, Wu D, Zhao Y, Li D, He D (2017). Knowledge and attitude of mothers regarding infant hearing loss in Changsha, Hunan province. China Int J Audiol.

[13] Lam MYY, Wong ECM, Law CW, Lee HHL, McPherson B (2018). Maternal knowledge and attitudes to universal newborn hearing screening: reviewing an established program. Int J Pediatr Otorhinolaryngol.

[14] Kaspar A, Newton O, Kei J, Driscoll C, Swanepoel DW, Goulios H (2017). Parental knowledge and attitudes to childhood hearing loss and hearing services in the Solomon Islands. Int J Pediatr Otorhinolaryngol.

[15] Alharbi MM, Almasri MS, Aldayel AY, Alkhonezan SM (2019). Parental knowledge, attitudes and practices towards Paediatric ear infections in Riyadh, Saudi Arabia: a quantitative study. Sultan Qaboos Univ Med J.

[16] Alshehri KA, Alqulayti WM, Yaghmoor BE, Alem H (2019). Public awareness of ear health and hearing loss in Jeddah, Saudi Arabia. S Afr J Commun Disord.

[17] Di Berardino F, Forti S, Iacona E, Orlandi GP, Ambrosetti U, Cesarani A (2013). Public awareness of ear and hearing management as measured using a specific questionnaire. Eur Arch Otorhinolaryngol.

[18] American Academy of Pediatrics (AAP) (2007). Year 2007 Position Statement: Principles and Guidelines for Early Hearing Detection and Intervention Programs. Pediatrics.

[19] World Health Organization. Childhood hearing loss [Internet]. Accessed at http://www.who.int/pbd/deafness/world-hearing-day/WHD2016_Brochure_EN_2.pdf.

[20] Sanju HK, Aggarwal K, Choudhary M, Yadav AK (2018). Knowledge and attitude of nurses towards infant hearing impairment in North India. Ind J Anat Sur Head Neck Brain.

